# Numerical model of aluminothermic reduction vessel preheating and charge heating with graphite heating rods

**DOI:** 10.12688/openreseurope.18586.1

**Published:** 2024-11-22

**Authors:** Sergey Semenov, Patrick Namy, Aditya Kale, Sello Tsebe

**Affiliations:** 1 SIMTEC, Grenoble, 38000, France; 2 MINTEK, Randburg, 2194, South Africa

**Keywords:** Reduction Vessel Preheating, Charge Heating, Graphite Heating Rods, Aluminothermic Reduction, Finite Element Method, COMSOL Multiphysics, Heat Transfer Modelling, Phase Change

## Abstract

**Background:**

The present work is conducted in the framework of the SisAl Pilot EU project, which aims to optimize silicon production in Europe by recycling materials and using carbon-emission-friendly technology. Silicon production experiments were conducted on laboratory and pilot scales in different types of furnaces, including reduction vessels used as chemical reactors for molten slag-metal mixtures. In addition to experimental work, process optimization also relies on numerical modelling.

**Methods:**

In this study, COMSOL Multiphysics® was used for numerical testing of new thermal and electrical designs of a reduction vessel by simulating its preheating and charge heating with three graphite heating rods powered by a three-phase alternating current transformer. A one-heating-rod design is also tested.

**Results and conclusions:**

The model predicts that available electrical equipment is sufficient for preheating the empty reduction vessel up to 1600°C in less than 4 h. Owing to the model, the geometry of the heating rod was optimized to maintain its temperature below 2500°C. However, it was found that a modification of the electrical equipment would be required to heat the vessel charge with the heating rods submerged into it.

## 1. Introduction

This work was conducted in the framework of the SisAl Pilot EU project, which is focused on demonstrating the possibility of metallurgical-grade silicon production at the pilot scale based on the aluminothermic reduction of silica. In comparison with the traditional carbothermic reduction of silica, the advantage of the proposed technology is its low CO2 emissions. As part of the project, the numerical modelling support of the experimental works was stipulated. One of the efforts focused on developing a numerical model of a new reduction vessel design proposed by MINTEK as a metallurgical reactor for the aluminothermic reaction between metal and slag. The objective of the present modelling work is to test the thermal and electrical performance of the new vessel design during preheating and aluminothermic reduction. The following hypotheses need to be tested: 1) the possibility of preheating the reduction vessel up to 1600°C in less than 4 h by using an existing MINTEK electrical equipment, while maintaining the temperature of graphite heating rods below 2500°C; 2) the possibility of continuous charge heating with help of three graphite heating rods submerged in the melt; 3) the risk of slag solidification during the aluminothermic reduction in the absence of external heating. In Section 2 and Section 3, the model geometry and numerical methods are described, respectively. Section 4 and Section 5 present the governing equations and material properties, respectively. In Section 6, numerical results are presented and discussed. Finally, Section 7 concludes the paper.

## 2. Problem geometry

**Figure 1.**
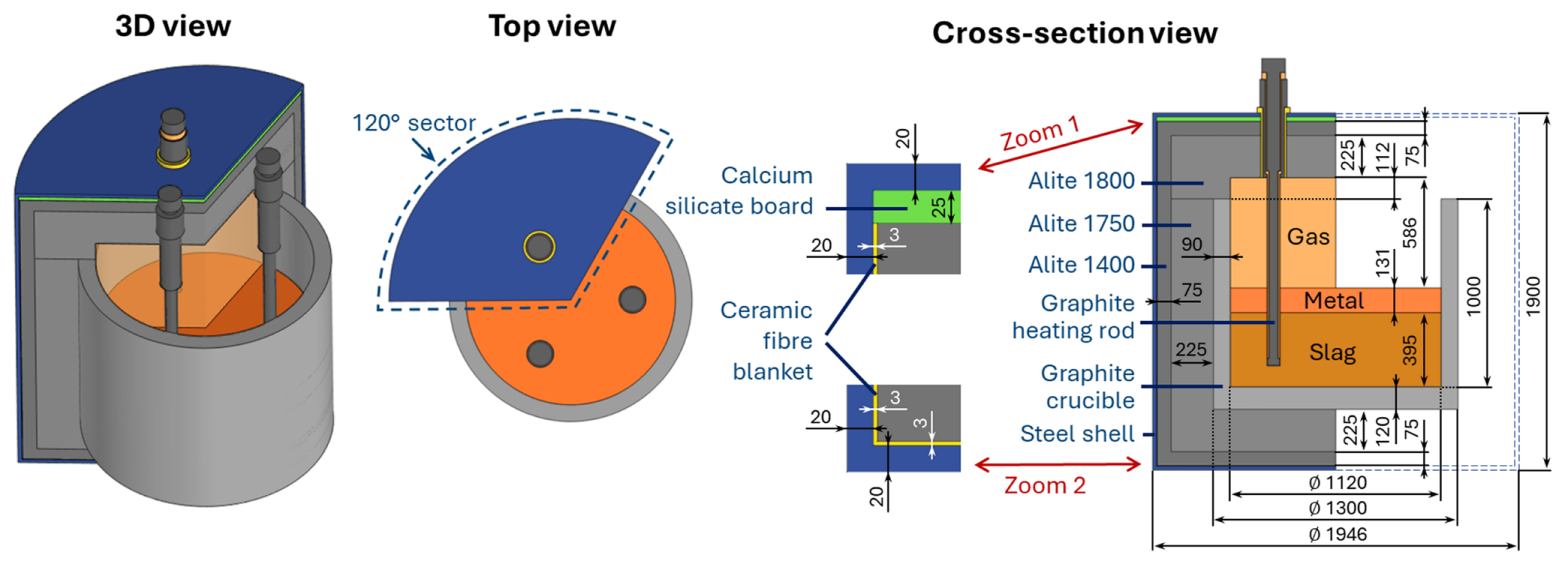
Modelled reduction vessel geometry and materials. Dimensions in mm.

**Figure 2.**
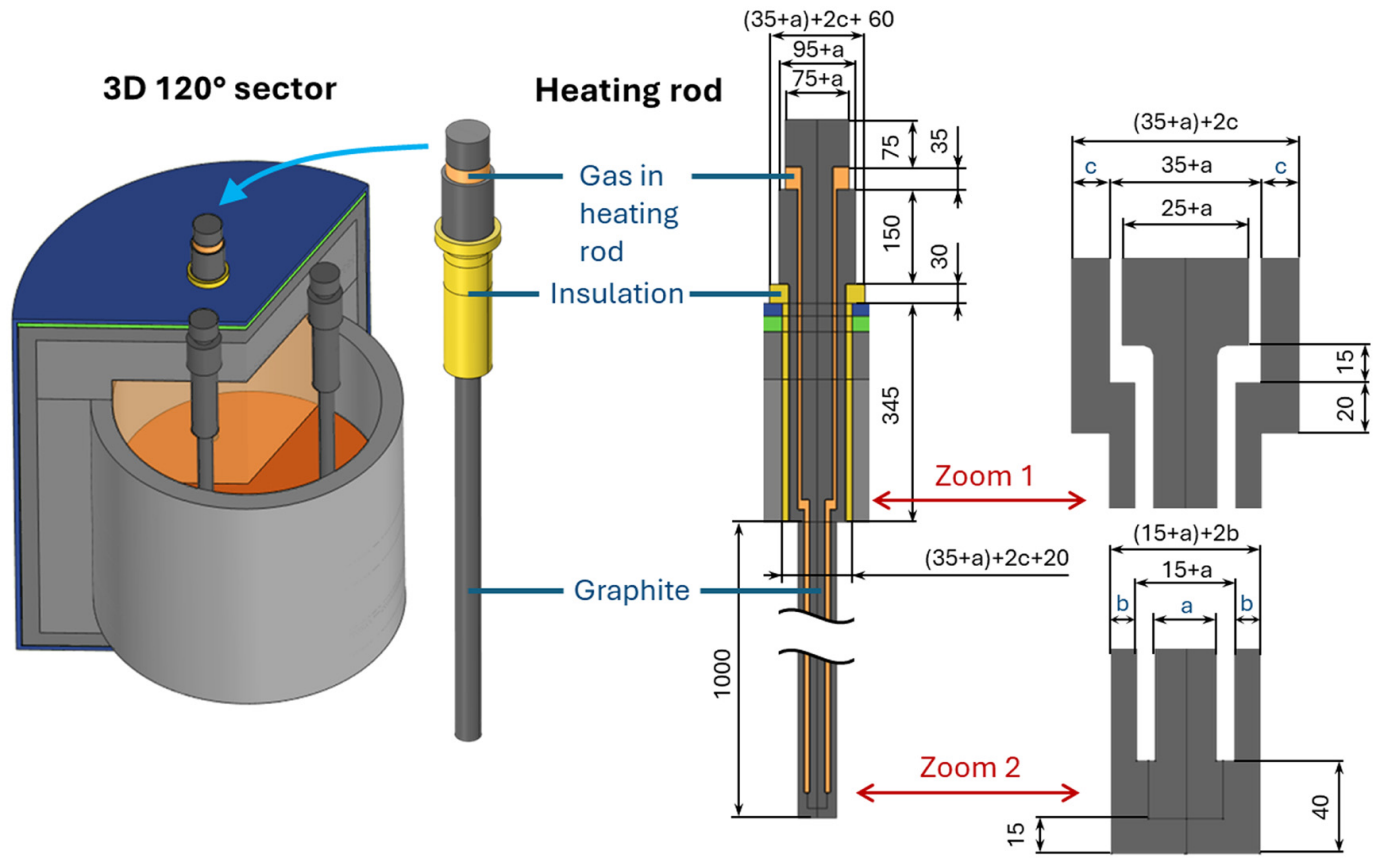
Heating rod geometry and materials. Dimensions in mm.

As shown in Figure 1, the reduction vessel has a cylindrical shape with 120° sector symmetry. It consists of a graphite crucible containing the vessel charge and gas, three graphite heating rods placed symmetrically around the axis of rotation, refractory layers, and the steel shell separated from the refractory by a calcium silicate board and a ceramic fiber blanket. The thermal part of the problem benefits from 120° sector symmetry, and therefore is resolved within only one 120° sector (see Figure 1). However, the electrical part of the problem has no sector symmetry because of the electrical contact between the three electrical phases via the electrically conducting vessel charge. Consequently, a complete 3D geometry was modelled for the electrically conducting parts of the vessel, including the vessel charge, crucible, and three heating rods. The heating rod geometry (see Figure 2) is based on existing equipment at Elkem, one of the project partners, and is given three adjustable parameters *a*, *b* and *c* to have some degrees of freedom for the problem optimization.

## 3. Methods

**Figure 3.**
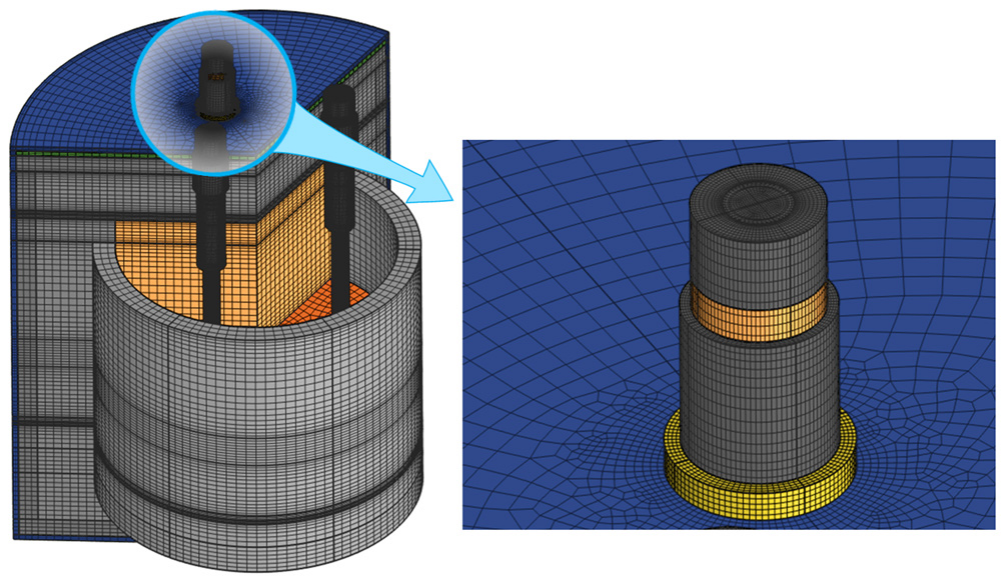
Spatial discretization mesh.

The problem was numerically solved using the finite element software COMSOL Multiphysics ^®^ version 6.0 ( www.comsol.com, a copyright license is obtained). The following modules have been used to set up the model physics: Heat Transfer in Solids and Fluids with phase change and convectively enhanced conductivity while fluid flow is not directly simulated, Surface-to-Surface Radiation, and Electric Currents to simulate the Joule effect in electrically conducting materials. Bidirectional coupling of all modules is present owing to multiple interdependencies via material properties. Each field was spatially discretized using linear Lagrange elements. Time integration is performed with a Backward Differentiation Formula (BDF) of orders 1 to 2. The computational domain is spatially discretized with a quadrilateral mesh that consists of 1 195 000 finite elements, as shown in Figure 3, resulting in 2 570 000 degrees of freedom. The computations were performed on two laptops with eight physical cores, an Intel processor with 64 GB RAM, and a workstation with 64 physical cores and 1 TB RAM.

## 4. Governing equations

### 4.1. Electric current equation

**Figure 4.**
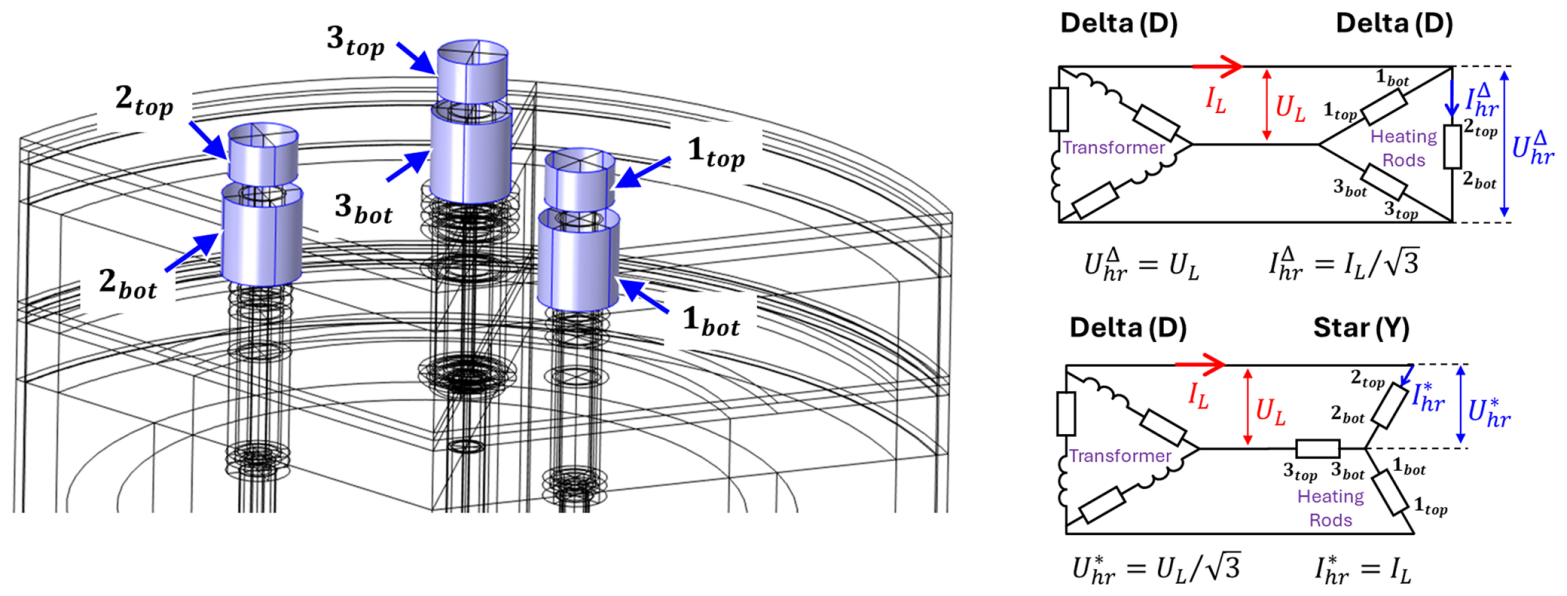
Heating rod terminals and connection types.

One of the requirements imposed by the reduction vessel design is the use of a 3-phase alternating current (AC) power source, such as an electrical transformer available at MINTEK. Thus, the present model implements AC electrical equations in the frequency domain formulation. The equations of current conservation are solved for all electrically conducting domains (metal, slag, crucible, and three heating rods):

∇⋅j=0,j=σE+jωD,

$$\text{E}=-\nabla V,\;\text{D}=\varepsilon_{0}\varepsilon_{r}\text{E},\;\omega =2\pi f$$

where **j** is the complex amplitude (or phasor) of the electric current density, **E** is the phasor of the electric field, *V* is the phasor of the electric potential, **D** is the phasor of the electric displacement field, *σ* is the electrical conductivity as a function of temperature, *f* is the alternating current frequency, *ω* is the angular frequency, *ε*_0_ is the vacuum permittivity, *ε*_r_ is the relative permittivity, and *j* = $\sqrt{-1}$ is the imaginary unit. The volume density of the heat source owing to the Joule effect is expressed as follows:

Q=12Re⁡(j⋅E*)

where asterisk E* denotes the complex conjugate of **E**, and Re( *Z*) is the real part of the complex number *Z*. The electrical boundary conditions on the heating rod terminals (see Figure 4) are defined by an electrical transformer that powers the vessel and by the type of 3-phase electrical connection of the heating rods: either delta (D) or star (Y) connection. Here, *U _L_* and *I _L_* are the effective line voltage and effective line current, $U_{hr}^{\Delta }$ and $I_{hr}^{\Delta }$ are the effective voltage and effective current through a heating rod in the delta configuration

$$U_{hr}^{\Delta }=U_{L},\;I_{hr}^{\Delta }\;=I_{L}/\sqrt{3}$$

and $U_{hr}^{*}$ and $I_{hr}^{*}$ are those in the star configuration:

$$U_{hr}^{*}=U_{L}/\sqrt{3},\;I_{hr}^{*}=I_{L}$$

The boundary conditions for the electric potential at the top terminals are as follows:

$$V_{i_{top}}=\sqrt{2}U_{hr}^{*}e^{j\varphi_{i}}=\frac{\sqrt{2}}{\sqrt{3}}U_{L}e^{j\varphi_{i}},\;i=1,2,3$$

with line phases *φ _i_* = *i* ⋅ 120°. For the bottom terminals, in case of star connection:

$$V_{i_{bot}}=V_{i_{bot}}^{*}=0,\;i=1,2,3$$

and in case of delta connection:

$$V_{i_{bot}}=V_{i_{bot}}^{\text{Δ}}=\frac{\sqrt{2}}{\sqrt{3}}U_{L}e^{j(\varphi_{i}+120{}^{\circ})},\;i=1,2,3$$

Electric insulation is assumed on other external boundaries of the electrically conducting domain:

j⋅n=0

### 4.2. Heat transfer with surface-to-surface radiation

**Figure 5.**
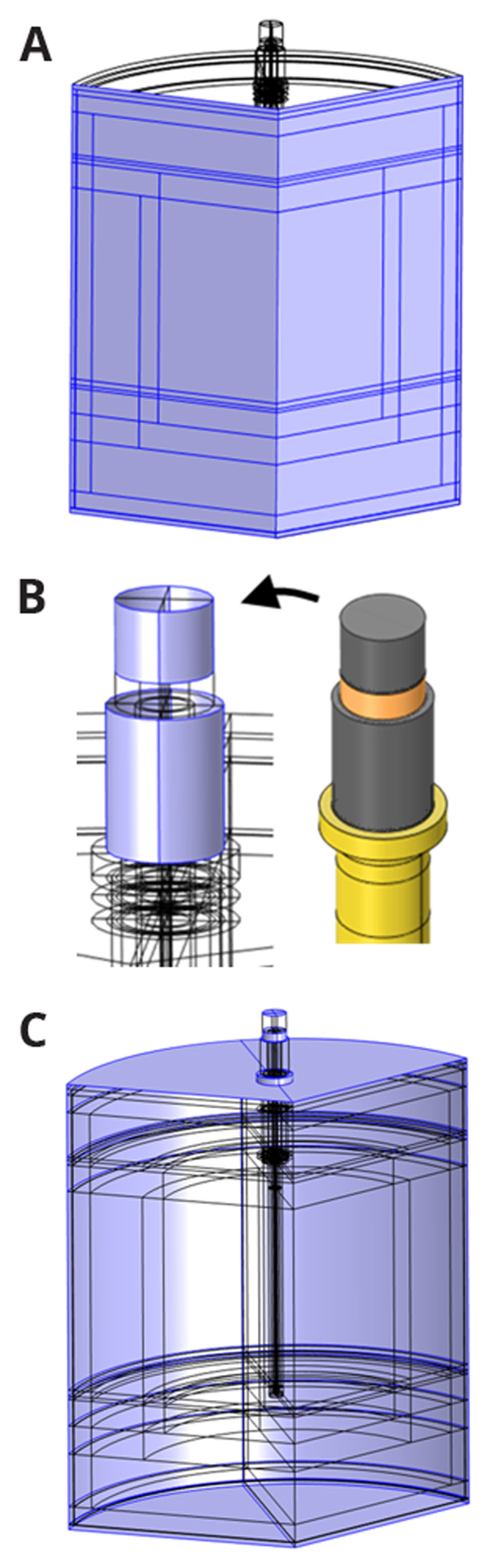
In blue: ( **a**) two symmetry planes, ( **b**) heating rod terminals, ( **c**) other external boundaries of the vessel.

The following heat equation is solved for all domains of the 120° sector, as shown in Figure 1.

$$\rho c_{p}^{\prime }\frac{\partial T}{\partial t}+\nabla \cdot \text{q}=Q,\;\text{q}=-\max(1,\text{Nu})\cdot k\nabla T$$

where *T* is the temperature, *t* is the time, *ρ* is the density, **q** is the convectively enhanced conductive heat flux, *k* is the thermal conductivity, $c_{p}^{\prime }$ is the isobaric specific heat capacity modified with the Apparent Heat Capacity method to account for phase changes, *Q* is the volume density of the heat source due to the Joule effect in electrically conducting materials and the heat loss in the crucible gas due to a protective gas flow with a modelled rate of 8 L/min. The conductivity *k* is convectively enhanced in the crucible gas with the Nusselt number Nu, which is computed according to an empirical model as a function of the temperature difference between the heating rod and crucible. A zero normal heat flux was imposed on the two symmetry planes, as shown in Figure 5 (a).

q⋅n=0

where **n** is the unit vector normal to the plane. As the electrical cables connected to the heating rods are supposed to be water-cooled, the ambient temperature *T _amb_* is imposed on the heating rod terminals, as shown in Figure 5 (b).

$$T=T_{amp}$$

On the other external surfaces of the vessel, as shown in Figure 5 (c), the heat flux owing to external natural convection is applied:

$$\text{q}\cdot \text{n}=h(T-T_{amp})$$

where **n** is the outward unit normal vector and *h* is the heat transfer coefficient computed according to several empirical models of external natural convection: one for a thin vertical cylinder and two models for the upper and lower horizontal plates. The temperature continuity on all material interfaces was applied as follows:

$$T_{up}=T_{down}$$

where the subscripts *up* and *down* denote opposite sides of the interface, with *up* corresponding to the positive direction of the normal vector **n**. The condition of the heat flux continuity/discontinuity at the material interfaces is written as follows:

$${\text{q}}_{\text{up}}\cdot \text{n}-{\text{q}}_{down}\cdot \text{n}=q$$

where *q* denotes the surface density of the interfacial heat source in W / m ^2^. In the presence of both the metal and slag, an interfacial heat source *q* at the metal-slag interface due to aluminothermic reduction is computed based on the reaction energy and reaction time. The interfaces adjacent to the gases (in the crucible and in the heating rod) participate in the surface-to-surface radiation and thus have the following interfacial heat source *q*:

$$q=\varepsilon (G_{rad}-\sigma_{SB}T^{4})$$

where *ε* is the hemispherical emissivity of the radiating surfaces, *σ _SB_* is the Stefan-Boltzmann constant, and *G* is the surface irradiance (surface density of the radiant flux in W/m ^2^, which arrives at the surface). A special 120° rotation symmetry condition was applied for the surface-to-surface radiation problem, which is not equivalent to a conventional zero-flux symmetry condition but allows radiant heat fluxes to pass through the symmetry planes.

## 5. Material properties and parameters

### 5.1. Graphite

For simplicity, the graphite model uses its porosity *ϕ*, including both open and closed pores, as a single adjustable parameter of the material, which, along with temperature *T*, defines all other properties of the material. We also assume that the porosity *ϕ* does not change with temperature *T*. The graphite density *ρ _gr_* is modelled as a linear function of the porosity *ϕ*:

$$\rho_{gr}(T,\phi )=(1-\phi )\rho_{C}(T)$$

where *ρ _C_* ( *T*) is the density of non-porous graphite, which is modelled as a function of temperature using the linearized thermal expansion coefficient:

$$\alpha_{V}(T)=-\frac{1}{\rho_{C}(T)}{\left(\frac{\partial \rho_{C}(T)}{\partial T}\right)}_{p}=\alpha_{V,\text{ref}}+{\left(\alpha_{V}\right)}_{T}^{\prime }(T-T_{\text{ref},\alpha })$$

and the theoretical density of non-porous graphite at a given temperature:

$$\rho_{C,\text{ref}}=2260\;\text{kg}/{\text{m}}^{3}\;\text{at}\;T_{\text{ref},\rho }=293\;\text{K}$$

**Figure 6.**
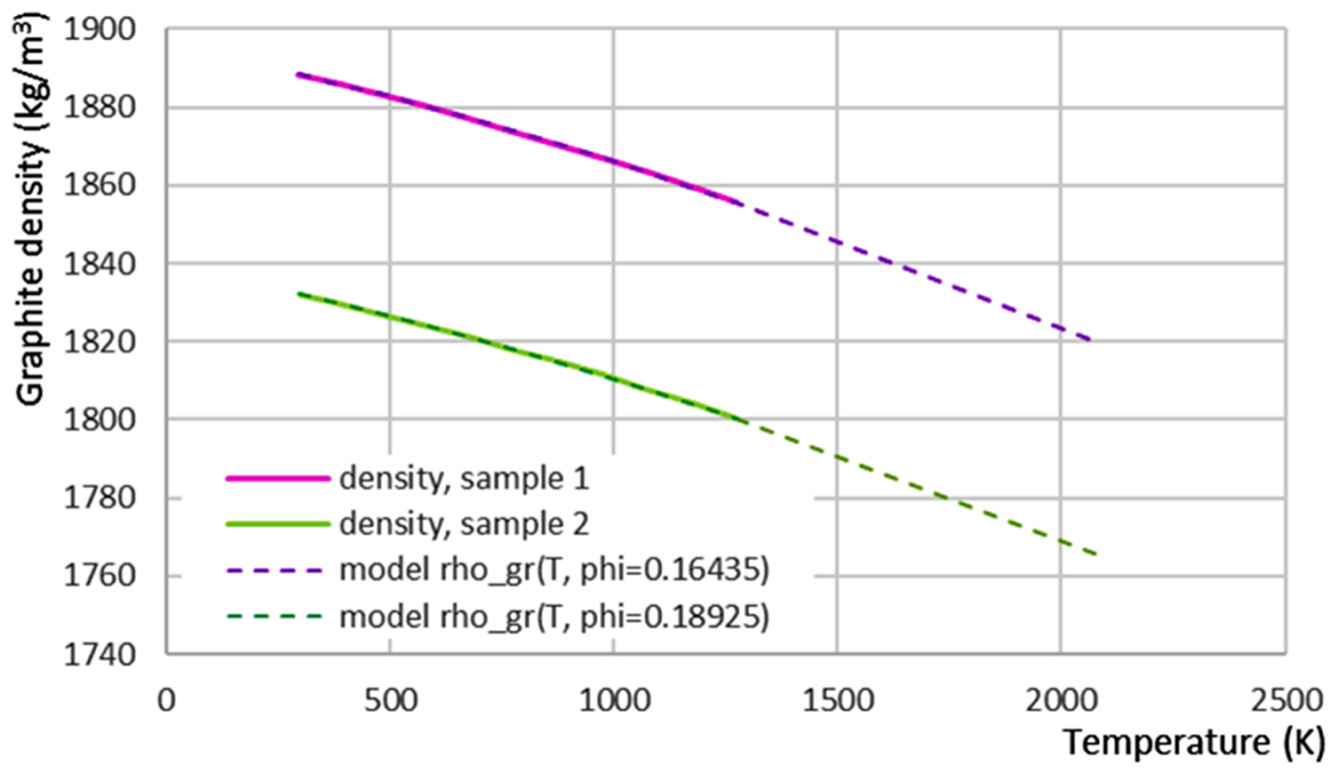
Fitting graphite density from literature ^1^ (samples 1 and 2) with the theoretical density model.

Assuming constant pressure *p = const* and integrating both sides of *α _V_* ( *T*) expression, we get:

$$\rho_{C}(T)=\rho_{C,\text{ref}}\;\text{exp}\left(-\alpha_{V}(T)\theta +{\left(\alpha_{V}\right)}_{T}^{\prime }\frac{\theta^{2}}{2}\right)$$

where *θ* = *T - T*_ref, *ρ*_. Using the above theoretical density model *ρ _gr_* ( *T,ϕ*) and fitting the value, slope, and second derivative of the temperature-dependent graphite density found in the literature ^1^ (see Figure 6), we obtain the theoretical porosity *ϕ* of the given material samples (see Figure 6), as well as the following thermal expansion constants:

$$\begin{array}{c} \alpha_{V,\text{ref}}=1.4508\times 10^{-5}\;{\text{K}}^{-1}\;\text{at}\;T_{\text{ref},\alpha }=293\;\text{K}\\ {\left(\alpha_{V}\right)}_{T}^{\prime }=6.9518\times 10^{-9}\;{\text{K}}^{-2} \end{array}$$

The specific heat capacity of graphite at a constant pressure *c _p,gr_* ( *T*) is assumed to be independent of the material porosity:

$$c_{p,gr}(T)=\{\begin{array}{ll} c_{p,\text{cup}}(T), & \;T\leq T_{1}=300\;\text{K}\\ c_{p,\text{app}}(T), & \;T>T_{1}=300\;\text{K} \end{array}$$

where *c _p,_*_app_( *T*) approximates literature data:

$$\begin{array}{l} c_{p,\text{app}}(T)=-5.8068\times 10^{-17}\cdot {\text{T}}^{6}+6.8603\times 10^{-13}\cdot T^{5}\\ \;-3.2774\times 10^{-9}\cdot T^{4}+8.1193\times 10^{-6}\cdot T^{3}\\ \;-1.1138\times 10^{-2}\cdot T^{2}+8.4443\;\cdot \;T-1.0091\times 10^{3} \end{array}$$

and *c*_*p*,cub_ is the cubic continuation of *c*_*p*,app_:

$$c_{p,\text{cub}}(T)={\frac{dc_{p,\text{app}}(T)}{dT}|}_{T=T_{1}}\;(T-T_{1})\frac{T^{2}}{T_{1}^{2}}+c_{p,\text{app}}(T_{1})\left(3-\frac{2T}{T_{1}}\right)\frac{T^{2}}{T_{1}^{2}}$$

where *T* is in K and *c _p,_*_app_ and *c*_*p*,cub_ are in J/kg/K. The thermal *k _gr_* and electrical conductivity *σ _gr_* of graphite are modelled as functions of its porosity *ϕ* using Landauer’s model of conductivity of a porous medium based on the effective medium percolation theory ^2^:

$$\begin{array}{c} k_{gr}(T,\phi )=f_{L}(k_{C}(T),\;k_{air}(T),\phi )\\ \sigma_{gr}(T,\phi )=f_{L}(\sigma_{C}(T),0,\phi ) \end{array}$$

where *k _C_* ( *T*) and *σ _C_* ( *T*) are the thermal and electrical conductivities of nonporous graphite, respectively, *k _air_* ( *T*) is the thermal conductivity of air trapped inside the pores, and *f _L_* is given by 2

$$\begin{array}{c} f_{L}(k_{s},k_{p},\phi )=\frac{1}{4}\left(s+\sqrt{s^{2}-8k_{s}k_{p}}\right)\\ s=k_{p}(3\phi -1)+k_{s}(2-3\phi ) \end{array}$$

where *k _s_* and *k _p_* are the solid and pore conductivity, respectively. The temperature dependencies of the non-porous graphite conductivities *k _C_* ( *T*) and *σ _C_* ( *T*) were obtained by fitting the graphite conductivities found in the literature ^1, 3^ with the theoretical models *k _gr_* ( *T, ϕ*) and *σ _gr_* ( *T, ϕ*), where the porosity *ϕ* is already known from density fitting. Several curves of *k _C_* ( *T*) and *σ _C_* ( *T*) obtained in this manner were then averaged, resulting in the following dependencies:

$$\begin{array}{c} k_{C}(T)=46758\;\text{W}/(\text{m}\cdot \text{K})\cdot {\left(T/1\;\text{K}+405\right)}^{-0.8543}\\ \sigma_{C}\left(T\right)=-7.1142\times 10^{-15}\cdot T^{6}+6.5797\times 10^{-11}\cdot T^{5}\\ \;-2.50415\times 10^{-7}\cdot T^{4}+5.2414\times 10^{-4}\cdot T^{3}\\ \;-0.67881\cdot T^{2}+509.18\cdot T+6743.35 \end{array}$$

where *T* is in K and *σ _C_* is in S/m. In the working range of vessel temperatures, the hemispherical emissivity of graphite *ε _C_* varies between 0.5 and 0.85 ^3^. The value of *ε _C_* = 0.5 is used in the current model as it corresponds to the worst-case scenario when the heating rod overheats faster. If we can avoid overheating for *ε _C_* = 0.5, we will also avoid it for larger values of *ε _C_*. The relative permittivity of graphite *ε _r,gr_* is taken as the volume average of non-porous graphite *ε _r,air_* = 15 and that of air *ε _r,air_* = 1:

$$\varepsilon_{r,gr}=(1-\phi )\varepsilon_{r,C}+\phi \varepsilon_{r,air}$$

Thus, two types of graphite, one for the crucible and the other for the heating rod, were assumed to be different only by their porosity. The porosity was calibrated by fitting the experimental data for another vessel at Elkem, which used the same graphite materials:

$$\phi_{\mathrm{crucible}}=0,\;\phi_{\mathrm{heating}\;\mathrm{rod}}=0.08$$

### 5.2. Slag and metal

The metal phase, which is initially pure Al, becomes an Al-Si-Ca alloy as it reacts with the slag. Similarly, initially SiO _2_-CaO slag becomes Al _2_O _3_-SiO _2_-CaO slag as it reacts with the metal. Thus, the slag and metal densities are computed as functions of the temperature *T* and composition *X _i_* (mole fraction of component *i*):

$$\rho (T,X_{i})=\sum_{i}\left[X_{i}M_{i}\right]/\left(\sum_{i}\left[X_{i}V_{m,i}(T)\right]+V^{EX}\right)$$

where *M _i_* and *V _m,i_* are the molar mass and molar volume ^4– 7^ of component *i*, *V ^EX^* is a corrective interaction term ^7^; *i* = Al, Si, Ca for the metal; and *i* = Al _2_O _3_, SiO _2_, CaO for the slag. The composition of the slag and metal is modelled as a function of the relative reaction extent *ξ _rel_* which equals 0 at the beginning of the process and 1 at the end of the reaction:

$$X_{i}=X_{i,init}+\xi_{rel}(X_{i,final}-X_{i,init})$$

For simplicity, it was assumed that the relative reaction extent *ξ _rel_* is a linear function of time.

$$\xi_{rel}=t/t_{r}$$

where *t _r_* is the user-defined reaction time. The initial *X _i,init_* and final *X _i,final_* compositions, as well as the reaction energy, were estimated using the software provided by the project partner SINTEF. This artificial neural network-based tool ^8, 9^ was trained on FactSage ^®^ data for the metal-slag system of interest. Other metal properties, such as thermal and electrical conductivity ^4^, dynamic viscosity ^10^, and heat capacity at constant pressure, were computed as for pure aluminum. The thermal conductivity of the slag was assumed to be constant and equal to 1 W/m/K. Other properties of the slag phase, such as heat capacity, enthalpy, viscosity, and electrical conductivity, were computed as functions of temperature *T* and composition *X _i_* according to the Ken Mills model ^11^.

### 5.3. Other materials

The heating rod gas was air and the crucible gas was argon. The densities were computed according to the ideal gas law. The air heat capacity was taken from the COMSOL Multiphysics ^®^ materials library, whereas the argon heat capacity was taken as a constant (520 J/kg/K). The thermal conductivity of both gases, as well as the viscosity of argon, which is needed for the Nu number computation, are modelled according to Sutherland’s law ^12^. The properties of the insulation material (see Figure 2) are experimentally unknown and, therefore, are arbitrarily selected: zero electrical conductivity, density 10 ^3^ kg/m ^3^, heat capacity 10 ^3^ J/kg/K, emissivity 0.85, and thermal conductivity 10 W/m/K. Structural steel properties from the material library were used for the steel shell. Its hemispherical emissivity was taken as for the iron oxide Fe _2_O _3_^13^. The properties of refractory materials are taken from material data sheets provided by the refractory supplier ^14^: Elite Cast 1400 INS for the external layer of the refractory, Elite ATB Cast 1750 for the internal body refractory, and Elite BA Cast 1800 INS for the internal roof refractory (see Figure 1). The calcium silicate board properties were obtained from the technical data sheet for the MB1000 Special material ^14^. The thermal conductivity (in W/m/K) was interpolated as a function of *T* (in K):

$$k_{MB1000}=9.7344\times 10^{-9}\cdot T^{2}+2.5007\times 10^{-6}\cdot T+2.0106\times 10^{-2}$$

Finally, the properties of the ceramic fiber blanket were modelled according to the specifications of Fibermax ^®^ Matte for a density of 130 kg/m ^3^. The heat capacity was estimated based on the composition.

## 6. Numerical results and discussion

### 6.1. Reduction vessel preheating and optimization of the heating rod geometry

**Figure 7.**
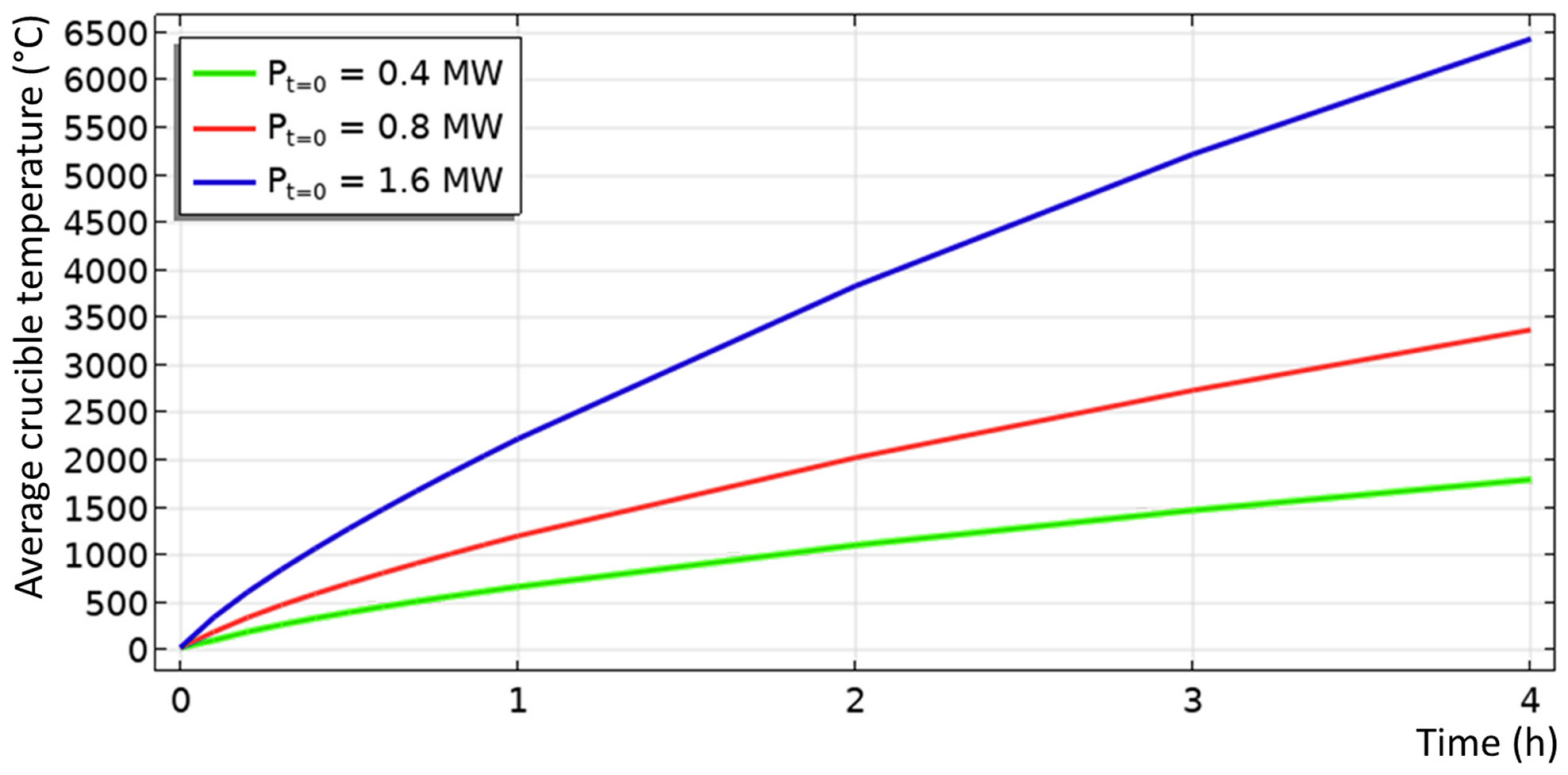
Computed average crucible temperature as function of time for different input power.

**Figure 8.**
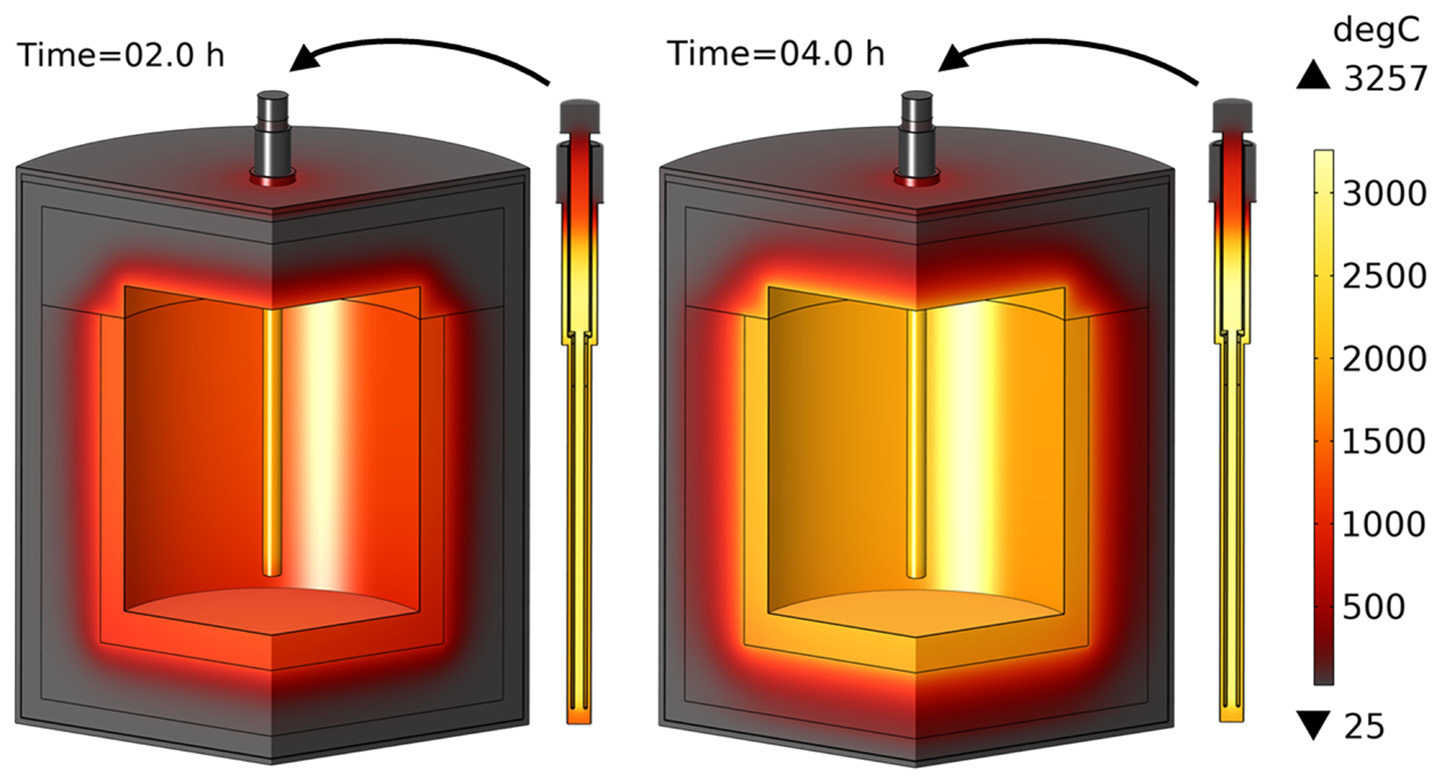
Temperature field for the input power of 0.4 MW and graphite emissivity of 0.5.

**Figure 9.**
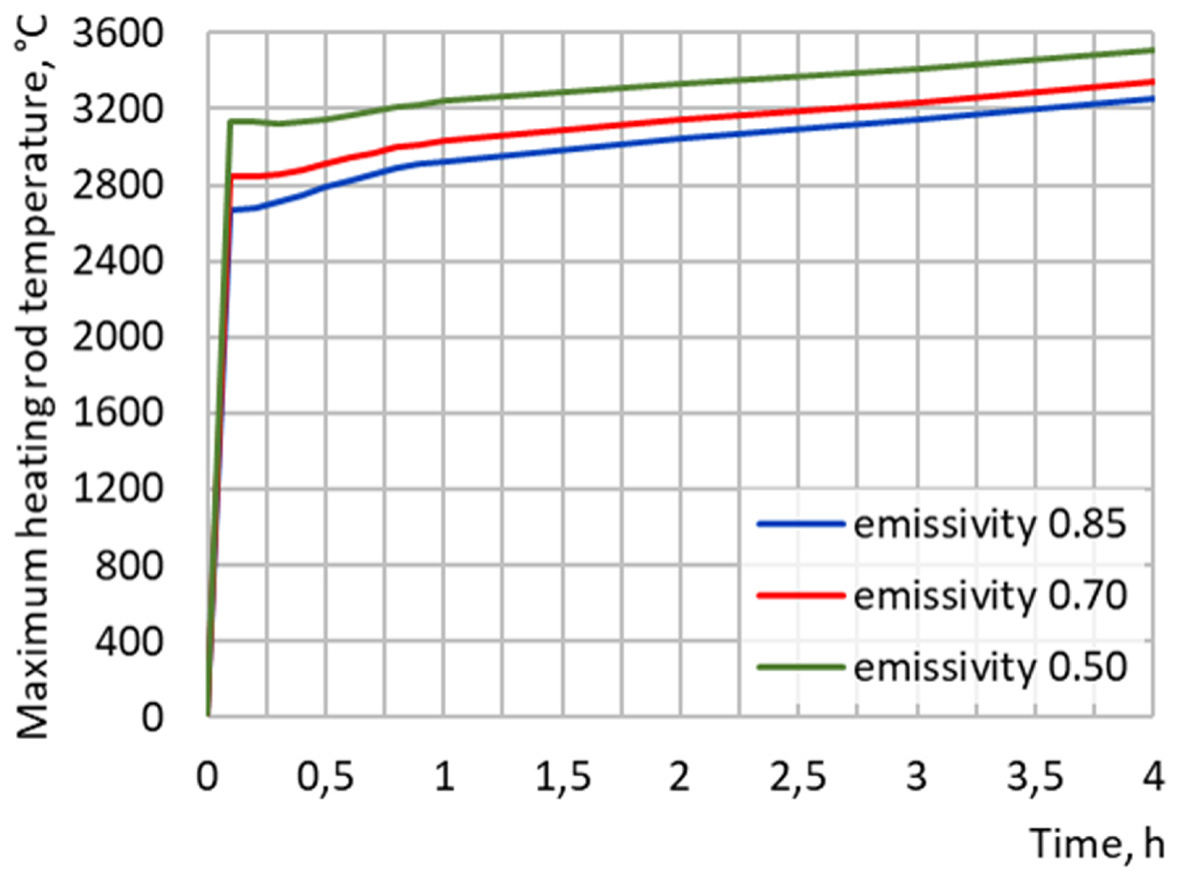
Maximum heating rod temperature at 0.4 MW input power for different graphite emissivity.

Numerical simulations show that input power above 0.4 MW will be sufficient to preheat the empty reduction vessel up to 1600°C in less than 4 h (see Figure 7). However, even at 0.4 MW input power, dangerous temperatures above 2500°C were reached (see Figure 8). Increasing the heating rod emissivity decreases its maximum temperature *T _hr,max_* (see Figure 9) but is not sufficient to be below 2500°C. The natural gas convection in the crucible or the heat loss due to a protective gas flow through the crucible have an almost negligible influence on *T _hr,max_*. Thus, the heating rod geometry was optimized by varying the three parameters *a*, *b*, *c* (see Figure 2) to satisfy 3 criteria:

1) The historical maximum power dissipation of the three heating rods should not exceed the maximum transformer power.

2) The average crucible temperature should reach 1600°C in less than 4 hours.

3) The maximum heating rod temperature *T _hr,max_* should not exceed 2500°C.

The result of such optimization shows that the delta (D) connection of heating rods (see Figure 4) should be avoided as it delivers too much power and overheats the rods, even for the minimum available transformer voltage. Instead, a star (Y) connection of the heating rods should be used. In this case, when a minimum line voltage *U _L_* of 105 *V* (as per available electrical equipment) is provided, the optimum geometry parameters are found to be

a=50mm,b=2mm,c=15mm

**Figure 10.**
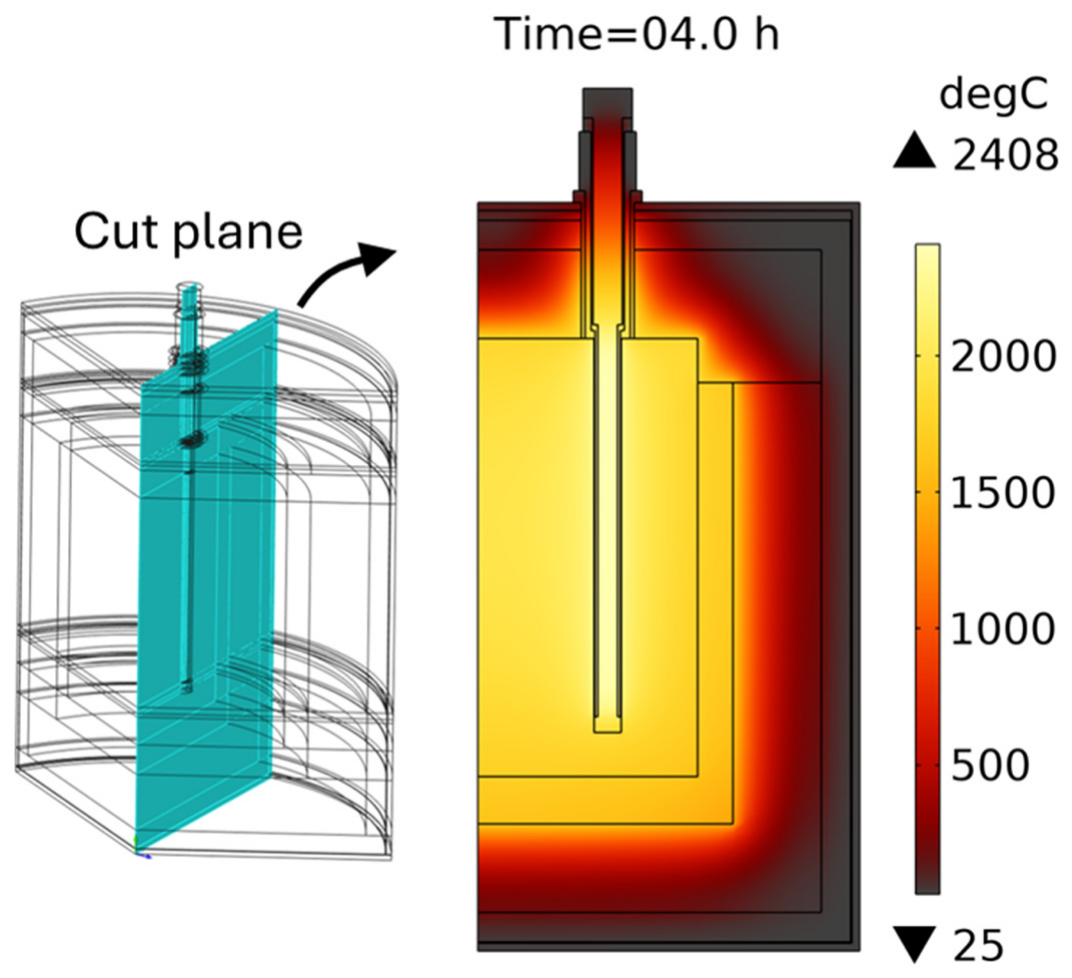
Temperature field after 4 hours of reduction vessel preheating with an optimized geometry of heating rods. Line voltage is 105 V.

With this heating rod geometry, the temperature field after 4 h of vessel preheating is shown in Figure 10: maximum heating rod temperature is below 2408°C, whereas average crucible temperature is above 1600°C. The transformer delivers between 337 and 410 kW of heating power, depending on the heating rod temperature at each moment in time. Note that in practice, in addition to the above three satisfied optimization criteria, there is also a mechanical strength requirement: the minimum thickness of the graphite parts must be greater than 1 cm. It can be seen that thickness *b* does not satisfy this criterion and becomes even thinner as the line voltage increases. Nevertheless, the optimized geometry of the rods will be used in further computations to study other thermal and electrical aspects of the reduction vessel design.

### 6.2. Charge heating with available transformer

**Figure 11.**
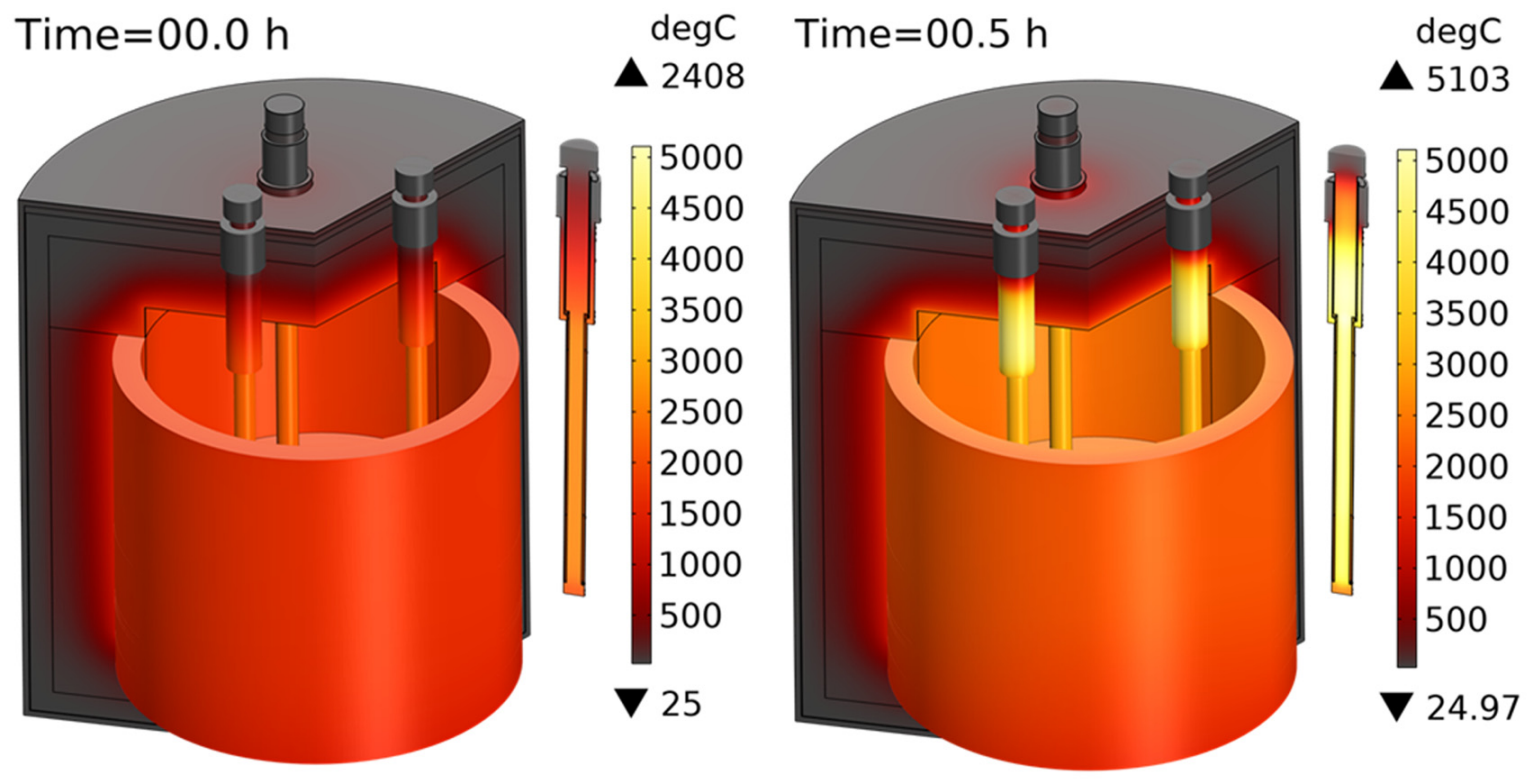
Temperature field when only slag is charged into the reduction vessel. Line voltage is 105 V.

**Figure 12.**
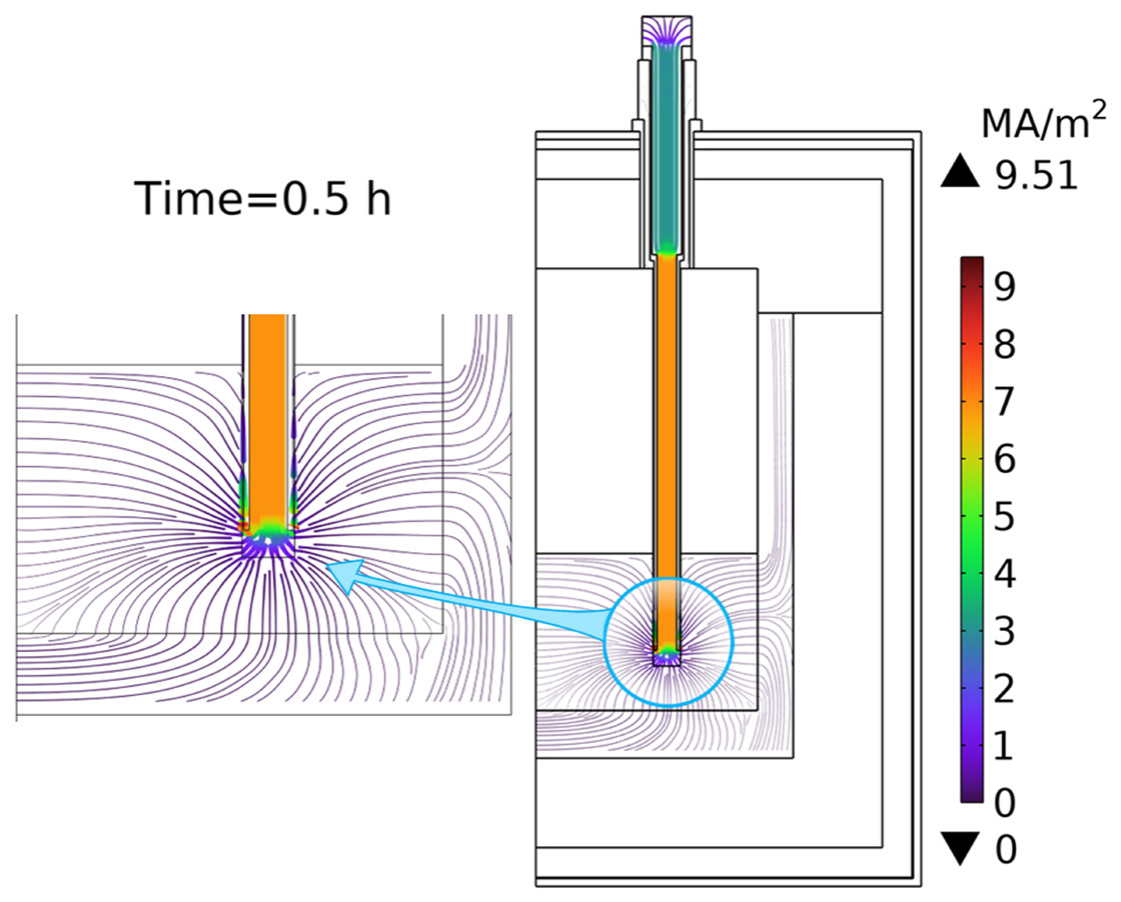
Map of the electric current density when only slag is charged into the vessel. Line voltage is 105 V.

Below, we present the results of the charge heating modeling with submerged heating rods powered by the available transformer: *U _L_* = 105 *V*. The previously computed preheated state served as the initial condition, initial slag temperature was 1650°C, initial metal temperature was 800°C. Two cases were investigated: **Case 1:** only the slag was charged, **Case 2:** both the slag and metal were charged. In **Case 1** there was no chemical reaction, and the vessel was heated only by an electrical heat source. Figure 11 shows the computed temperature fields before and after 30 min of heating. As one can see, the temperature inside heating rods rises to 5103°C because of high electric currents passing through them (see Figure 12). The current goes down the internal graphite cylinder of the heating rod, and then, instead of going up through the external graphite cylinder, it prefers to pass through the slag volume and graphite crucible towards the two other graphite rods, which are also submerged into the slag. Apparently, this path is less resistive and creates a short circuit between heating rods. The effective line current *I _L_* approaches 10 ^4^ A, which is two times higher than the allowed transformer’s maximum, and therefore, is dangerous for electrical equipment. In addition, as the slag temperature increases owing to heating, its electrical conductivity increases, which results in further growth of the electric current over time. To conclude **Case 1**, the presence of slag in the vessel resulted in a short circuit between heating rods, in dangerous transformer currents, and in graphite overheating above 2500°C. A similar conclusion was made for **Case 2** when both slag and metal were charged. The heat produced by the reaction and the high conductivity of the metal did not improve this situation.

### 6.3. Charge heating with reduced line voltage

**Figure 13.**
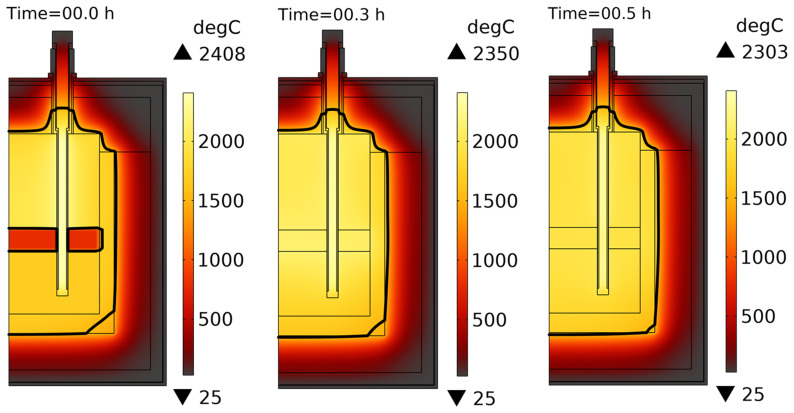
Temperature field evolution when both slag and metal are charged into the reduction vessel. Line voltage is 40 V. Thick black line represents the slag melting temperature isoline (1540°C).

Let us consider the worst-case configuration in which both the slag and metal are charged into the vessel. In this case, we can try to reduce the effective line voltage *U _L_* to provide a reasonable electric power input and avoid the above-mentioned problems of excessive current and graphite overheating. The duration of the aluminothermic reduction was assumed to be 20 min, during which the reaction energy was released at the slag-metal interface. By studying the influence of the line voltage *U _L_* on the modelling results, it was found that a line voltage below 40 V is acceptable for vessel charge heating with three heating rods submerged in the melt. Figure 13 shows the resulting evolution of the vessel temperature field. The results show that the heating rod temperature decreases with time as the electrical input power is below that provided during the preheating stage. The crucible temperature slowly increased with time owing to the joint contribution of the electrical and chemical (≈ 890 kW) power input. If such a transformer setting with *U _L_* = 40 V is possible, it would result in approximately 180 kW of electrical power input, which would be enough to compensate for heat losses and to maintain the charge above its melting point over a long period of time to maximize the chemical reaction output. In this hypothetical vessel configuration, the heating rod temperature remained below the maximum acceptable graphite temperature of 2500°C, and the effective line current *I _L_* did not exceed the allowed maximum provided by the transformer.

### 6.4. Aluminothermic reduction with no external heating

**Figure 14.**
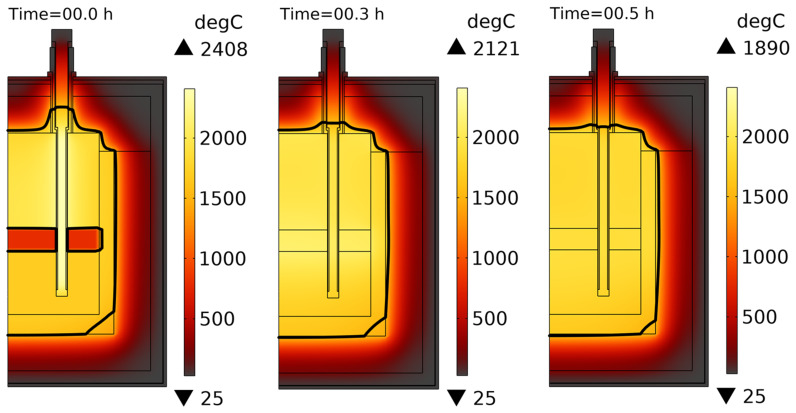
Temperature field evolution when both slag and metal are charged into the reduction vessel with no electrical power input. Thick black line represents the slag melting temperature isoline (1540°C).

The results of modelling the situation when no electrical power was provided are shown in Figure 14. In this case, the maximum temperature in the vessel reduced faster with time than in the case with nonzero electrical power. Nevertheless, the aluminothermic reduction power alone is sufficient to maintain the melt in a liquid state for at least 30 min.

### 6.5. Reduction vessel preheating and charge heating with a single heating rod

**Figure 15.**
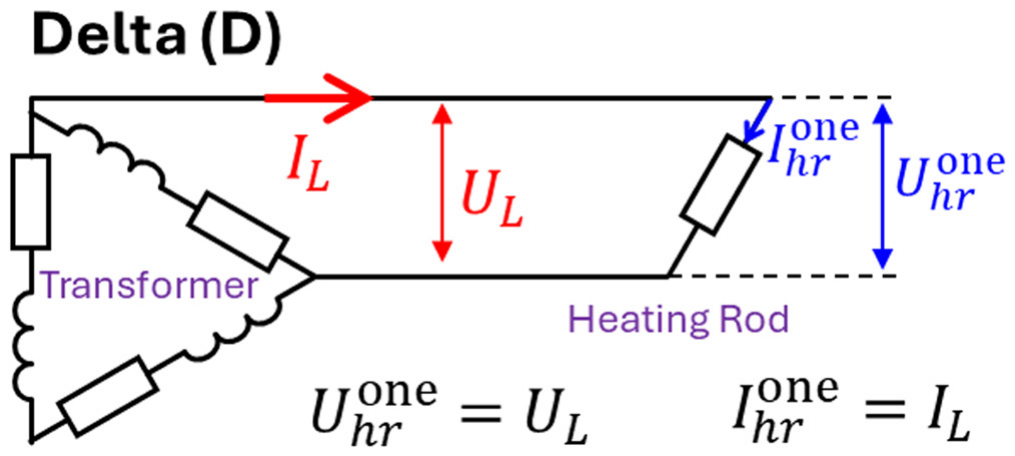
One heating rod connection.

**Figure 16.**
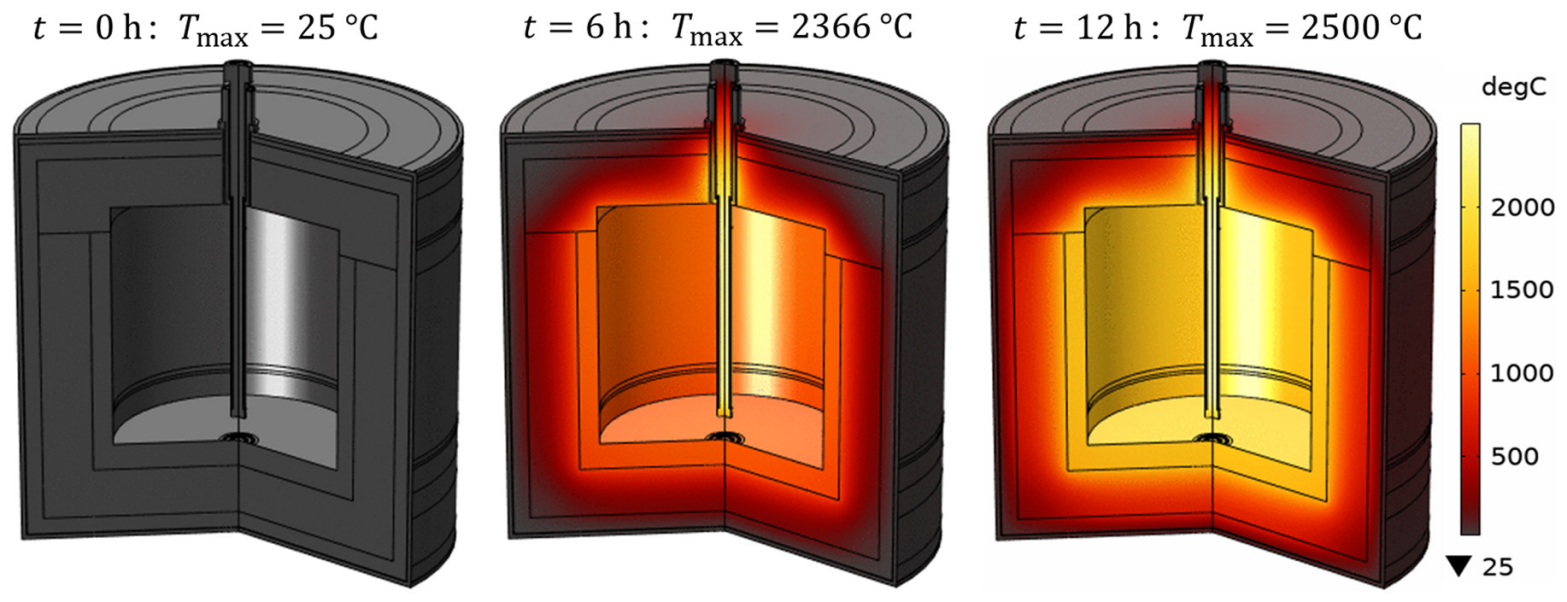
Time evolution of the temperature field during vessel preheating with a single heating rod at *U _L_* = 70 V.

**Figure 17.**
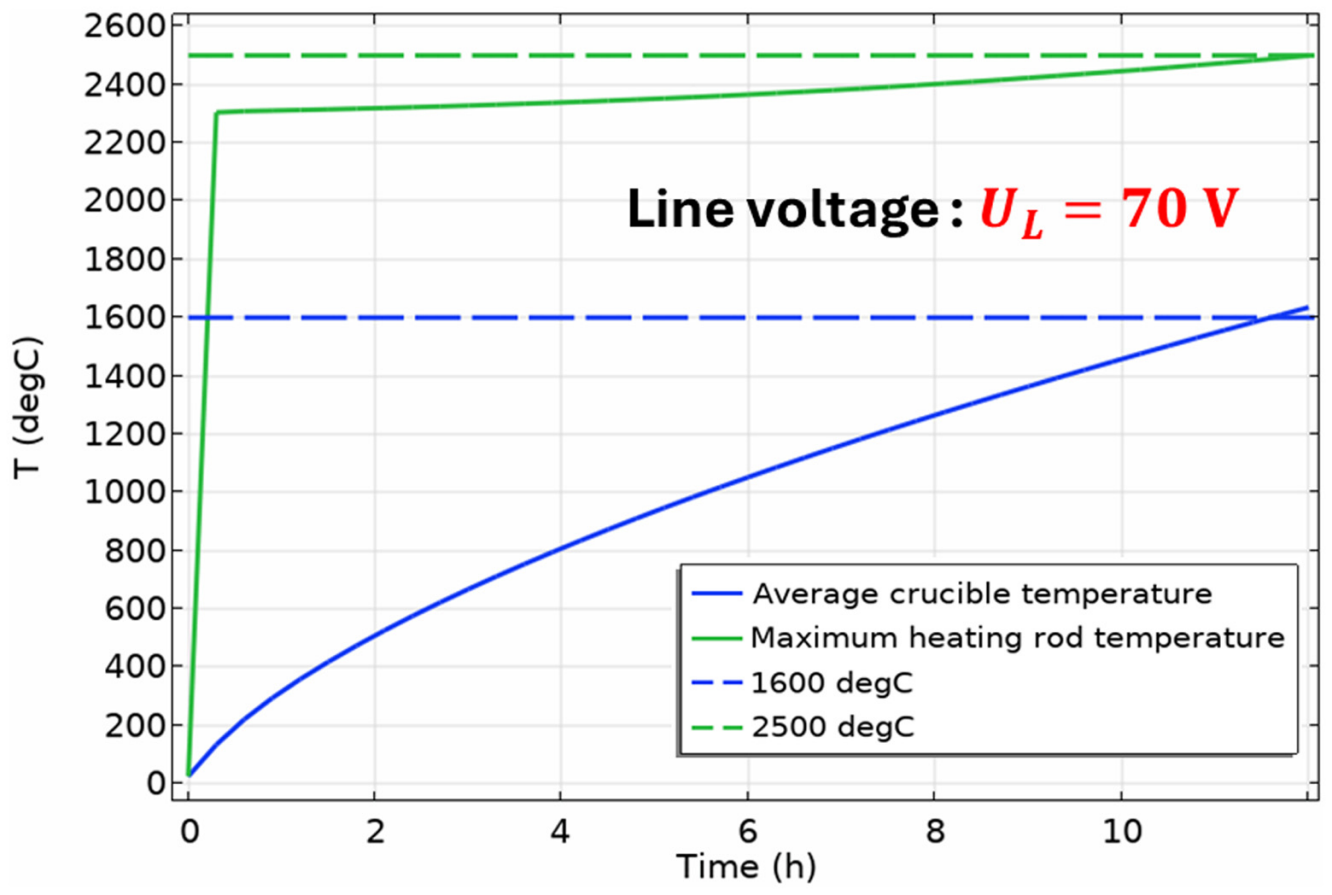
Time evolution of the average crucible temperature and of the maximum heating rod temperature in a single-heating-rod configuration of the vessel.

In the above results, we have seen that heating the vessel charge with three submerged heating rods is problematic because the electrically conducting slag and metal create a short circuit between the rods, which results in thermal and electrical damage to the reduction vessel and transformer. Therefore, in this section, a configuration with a single heating rod submerged in the melt at the center of the vessel is studied, as it excludes the possibility of a short circuit between the three electrical phases. The electrical circuit was modified as shown in Figure 15. The analysis shows that avoiding the transformer damage, the power dissipated in a single heating rod should not exceed 50% of the nominal transformer power for a given line voltage *U _L_*. Numerical testing of the feasibility of vessel preheating with a single heating rod gives an optimum line voltage of *U _L_* = 70 V, which is below the possible minimum of 105 V as per the available electrical equipment. According to the model, a line voltage of 70 V would result in an optimum time for vessel preheating up to 1600°C with heating rod temperature not exceeding 2500°C (see Figure 16 and Figure 17). However, it still requires 12 h of preheating instead of the desired 4 h, because the power provided by the transformer in this case is approximately 186 kW. Thus, preheating the reduction vessel with one heating rod can be considered impractical and unrealizable with the currently available equipment at MINTEK.

**Figure 18.**
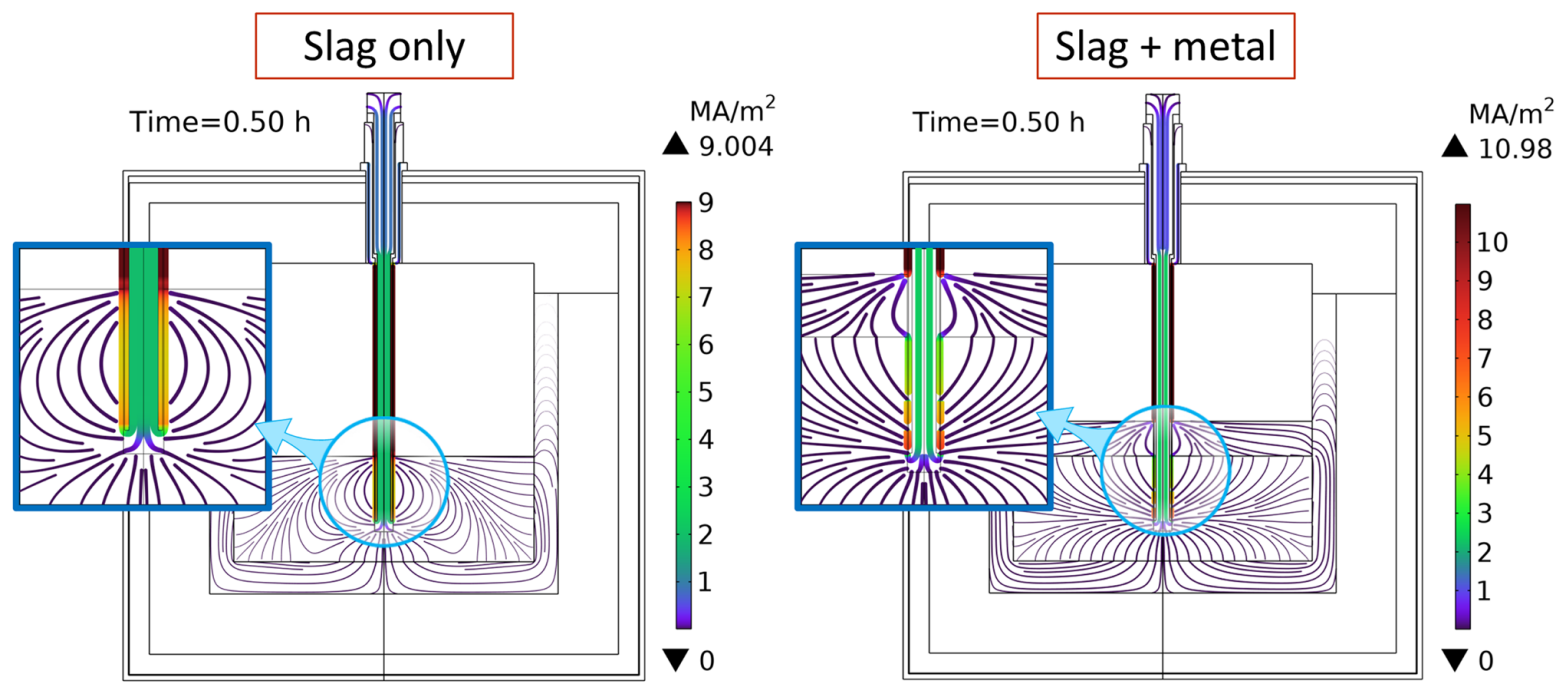
Map of electric current density when heating rod is submerged into the vessel charge at *U _L_* = 70 V.

Modelling of charge heating with a single heating rod submerged into the melt shows that further reduction of line voltage is needed to avoid thermal damage to the heating rod. In the case of only charged slag, a line voltage *U _L_* below 65 V is required, whereas in the case of both charged slag and metal, a line voltage *U _L_* below 48 V would be safe. The need for voltage reduction can be explained by the increase in the line current *I _L_* when the heating rod is submerged in the molten charge. This occurs because the electrical resistance between the heating rod terminals is reduced owing to the nonzero electrical conductivity of the slag and metal layers. The numerical model demonstrates how the electric current bypasses the submerged portion of the heating rod via the electrically conducting charge volume, as shown in Figure 18. Thus, a short circuit is created between the lower and upper submerged parts of the external graphite cylinder of the heating rod, which increases the power input and eventually overheats the heating rod.

## 7. Conclusions

The present numerical model predicts that available electrical equipment is sufficient for preheating the empty reduction vessel up to 1600°C in less than 4 h. Owing to the model, the geometry of the heating rod was optimized to maintain its temperature below 2500°C. However, it was found that the electrical function of the reduction vessel is strongly affected by the presence of the vessel charge, as both slag and metal are electrically conducting materials. The configuration where three heating rods are submerged into the molten charge is not acceptable, as it leads to a short circuit between heating rods, to an effective line current *I _L_* exceeding the allowed maximum provided by the transformer, and to the graphite overheating above the maximum acceptable temperature of 2500°C. The reduction of the line voltage down to 40 V was found to be sufficient to avoid electrical and thermal damage to the reduction vessel in the configuration with three heating rods submerged into the melt. Similarly, in the single-heating-rod configuration, vessel preheating is possible when the line voltage *U _L_* does not exceed 70 V, slag heating is possible when *U _L_* < 65 V, and slag and metal heating is possible when *U _L_* < 48 V. Otherwise, thermal damage to the graphite heating rod is unavoidable. Because reducing the line voltage below 105 V might not be possible, as it requires the replacement of the existing transformer, a numerical study with no electrical power input has been performed. This shows that the aluminothermic reduction power alone is sufficient to maintain the melt in a liquid state for at least 30 min. If electrical insulation of heating rods from the melt could be found, it would result in normal functioning of the electrical circuit, even with the original transformer settings. However, in this case, overheating of the graphite rods might be possible during the aluminothermic reduction process due to the reaction heat. Thus, according to the model, switching off the electrical power supply is recommended for the duration of the aluminothermic reduction process when the heating rods are submerged in the melt.

## Ethics and consent

Ethical approval and Consent were not required.

## Data Availability

Repository: Zenodo. Dataset title: “Refractory materials used in the numerical model of an aluminothermic reduction vessel” https://doi.org/10.5281/zenodo.13880179 ^14^. This project contains the following underlying data: Data file 1: “Elite ATB Cast 1750.png” (Data sheet for the properties of Elite ATB Cast 1750 material) Data file 2: “Elite BA Cast 1800 INS.png” (Data sheet for the properties of Elite BA Cast 1800 INS material) Data file 3: “Elite Cast 1400 INS.png” (Data sheet for the properties of Elite Cast 1400 INS material) Data file 4: “MB1000 Special.png” (Data sheet for the properties of MB1000 Special material) Data are available under the terms of the Creative Commons Attribution 4.0 International licence. No extended data associated with this article.
