# Supplementary material for: Numerical model of aluminothermic reduction vessel preheating and charge heating with graphite heating rods
Source: Open Res Eur. 2024 Nov 22;4:253. [Version 1] doi: 10.12688/openreseurope.18586.1 (PMC12749567; doi:10.12688/openreseurope.18586.1)
Supplement: Peer review history

# Reviewer report: Reviewer response for version 1

**DOI:** 10.21956/openreseurope.20105.r62728

**Published:** 2025-12-29

**Authors:** Mickaël Antoni

**Affiliations:** 1 Aix-Marseille Université, Marseille, France

This article presents numerical investigations of vessel designs intended for high-temperature chemical reactors, involving both electrical and thermal transfer processes. It aims to help the understanding of experimental conditions needed for running conducted with a pilot scale facility for the production of metallurgical-grade silica via aluminothermic reduction, a method that offers an improved carbon footprint (particularly in terms of CO₂ emissions) compared to traditional carbothermic techniques.

 The main objective is to optimize high-temperature processes using models and simulations performed with COMSOL Multiphysics, accounting material safety constraints, electrical supply characteristics and energy balance. Three specific goals are pursued:

 ·         To verify whether a 4-hour preheating phase can bring the bath of preheat the empty vessel up to 1,600 °C.

 ·         To assess the feasibility of regulating the heating of the molten bath using three graphite electrodes.

 ·         To analyze the risks associated with the formation of solid slag during the aluminothermic reduction phase (slag solidification).

 The article is clear and well-written. The illustrations are consistent with the provided explanations. The system geometry, including the structure and dimensions of the crucible and graphite electrodes, is described in detail. The numerical method is presented concisely yet sufficiently: the COMSOL modules used to describe the physical properties of the materials phenomena are mentioned, as are the discretization scheme (linear Lagrangian finite elements) and the numerical time integration scheme (BDF).

 The core section of the article focuses on modeling the entire experimental facility, including the crucible structure, graphite electrodes, and molten bath. The description of the equations used to model electrical and radiative phenomena is appropriate and detailed. Particular attention is given to the physicochemical properties of the materials, especially those of graphite and the emissivity value of the heating rods.

 Simulations were conducted for four distinct scenarios, including reduced voltages and the use of a single graphite rod. The results demonstrate that the available equipment can indeed achieve 1,600 °C in less than 4 hours while avoiding exceeding 2,500 °C at the vessel level. However, the three-electrode setup in the presence of a molten bath poses a risk of short circuits, potentially compromising both the power source and the vessel structure. In contrast, the single-rod configuration allows risk limitation within specific voltage ranges.

 The authors mention the limitations of the models, particularly the use of empirical descriptions for convective phenomena and the calculation of the heat transfer coefficient. A turbulent CFD model would substantially improve our understanding of convective heat transfer and the transport of chemical species within the molten bath. The properties of the graphite rods insulation were chosen arbitrarily but as realistically as possible. Figure 13 indicates a potential risk of exceeding the working temperature of refractory materials around the rods insulation. Analyzing this aspect of the thermal vessel design in more detail would enable the prediction of suitable thermal properties and dimensions of the insulation material. Another simplifying assumptions was that the aluminothermic reaction rate remained constant for the full 20 minutes of the reaction, resulting in a constant exothermic heat power. In perspective, a more realistic decaying reaction rate could be implemented based on the depletion of reacting species, for example. These points provide interesting openings and perspectives for future research.

 Despite these limitations, this article remains robust and offers a valuable contribution to the use of low-carbon processes, a major challenge in metallurgy. The results and perspectives presented make this article relevant to the scientific community.

Is the study design appropriate and does the work have academic merit?

Yes

Is the work clearly and accurately presented and does it cite the current literature?

Partly

If applicable, is the statistical analysis and its interpretation appropriate?

Not applicable

Are all the source data underlying the results available to ensure full reproducibility?

Yes

Are the conclusions drawn adequately supported by the results?

Yes

Are sufficient details of methods and analysis provided to allow replication by others?

Yes

Reviewer Expertise:

Simulation of heat and mass transfer mechanisms (CFD), dispersed systems (emulsions, aerosols), corium-concrete interaction, instrumentation.

I confirm that I have read this submission and believe that I have an appropriate level of expertise to confirm that it is of an acceptable scientific standard.

# Reviewer report: Reviewer response for version 1

**DOI:** 10.21956/openreseurope.20105.r48721

**Published:** 2025-01-10

**Authors:** Kagan Benzesik

**Affiliations:** 1 Istanbul Technical University, Istanbul, Turkey

The article *"Numerical Model of Aluminothermic Reduction Vessel Preheating and Charge Heating with Graphite Heating Rods"* investigates the optimization of reduction vessels used for sustainable silicon production. Using COMSOL Multiphysics, the authors model the thermal and electrical performance of vessels during preheating and charge heating processes, focusing on graphite heating rods powered by a three-phase transformer. The study finds that preheating the vessel to 1600°C within 4 hours is feasible with current equipment, but submerged heating rods cause short circuits, transformer overloading, and graphite overheating. The model suggests that reducing line voltage to 40V or insulating the rods could mitigate these issues.

 The research contributes to the SisAl Pilot EU project, promoting low-carbon aluminothermic reduction methods. 

 This article presents a well-structured and impactful study that combines rigorous numerical modeling with practical applications in sustainable metallurgical processes. The insights gained from optimizing reduction vessel designs and identifying challenges like heating rod overheating or transformer limitations offer valuable guidance for both researchers and industry professionals. By addressing the suggested improvements—such as enhancing readability, contextualizing findings within broader research, and proposing future directions—the article has the potential to make a significant contribution to the field. Overall, the work stands as a promising advancement in the pursuit of low-carbon metallurgical technologies and aligns effectively with global sustainability goals.

Is the study design appropriate and does the work have academic merit?

Yes

Is the work clearly and accurately presented and does it cite the current literature?

Partly

If applicable, is the statistical analysis and its interpretation appropriate?

Not applicable

Are all the source data underlying the results available to ensure full reproducibility?

Yes

Are the conclusions drawn adequately supported by the results?

Yes

Are sufficient details of methods and analysis provided to allow replication by others?

Yes

Reviewer Expertise:

metallothermic reduction, combustion synthesis, self propagating high temperature synthesis

I confirm that I have read this submission and believe that I have an appropriate level of expertise to confirm that it is of an acceptable scientific standard.
